# Carbohydrate Nutrition and Team Sport Performance

**DOI:** 10.1007/s40279-015-0399-3

**Published:** 2015-11-09

**Authors:** Clyde Williams, Ian Rollo

**Affiliations:** School of Sport, Exercise and Health Sciences, Loughborough University, Loughborough, Leicestershire, England, LE11 3TU UK; Gatorade Sports Science Institute, Leicester, UK

## Abstract

The common pattern of play in ‘team sports’ is ‘stop and go’, i.e. where players perform repeated bouts of brief high-intensity exercise punctuated by lower intensity activity. Sprints are generally 2–4 s long and recovery between sprints is of variable length. Energy production during brief sprints is derived from the degradation of intra-muscular phosphocreatine and glycogen (anaerobic metabolism). Prolonged periods of multiple sprints drain muscle glycogen stores, leading to a decrease in power output and a reduction in general work rate during training and competition. The impact of dietary carbohydrate interventions on team sport performance have been typically assessed using intermittent variable-speed shuttle running over a distance of 20 m. This method has evolved to include specific work to rest ratios and skills specific to team sports such as soccer, rugby and basketball. Increasing liver and muscle carbohydrate stores before sports helps delay the onset of fatigue during prolonged intermittent variable-speed running. Carbohydrate intake during exercise, typically ingested as carbohydrate-electrolyte solutions, is also associated with improved performance. The mechanisms responsible are likely to be the availability of carbohydrate as a substrate for central and peripheral functions. Variable-speed running in hot environments is limited by the degree of hyperthermia before muscle glycogen availability becomes a significant contributor to the onset of fatigue. Finally, ingesting carbohydrate immediately after training and competition will rapidly recover liver and muscle glycogen stores.

## Key Points

**Table:** 

Repeated brief periods of variable speed running lower muscle glycogen stores.
Lowered muscle glycogen stores reduces performance during subsequent variable speed running.
A high carbohydrate diet during recovery from prolonged periods of variable speed running restores muscle glycogen and subsequent performance.

## Introduction

All athletes are part of teams whether as track athletes, swimmers or football players. However, when describing team sport performance we usually mean teams in which players depend on each other to out-score their opponents. For example, in football, soccer, field and ice hockey, rugby, basketball and lacrosse, success depends on the mutual cooperation of players to score more goals/points than the opposition.

Above and beyond the nutritional requirements to sustain good health, the additional nutritional needs of players vary according to the demands of their sport and their playing positions within their sport. In addition, team sport players represent the full spectrum of body shapes and sizes from the large body mass of football linemen through to lean soccer players. Ideally, nutritional support should be customised to meet the needs of the individual player to ensure that they cope with training and competition [1]. Thereafter, their performance in competition depends on a range of intrinsic characteristics, such as skills, psychology and external influences such as the quality of the opposition and environmental conditions.

The common pattern of play in ‘team sports’ is ‘stop and go’, i.e. where players perform repeated bouts of brief high-intensity exercise punctuated by lower intensity activity. For example, soccer players sprint to tackle an opponent or gain possession of the ball, dribble it before passing and then jog into position to support an attack or defence. These sprints are rarely longer than 3–4 s followed by recovery of no more than several seconds before players are in action again [2]. In addition, some team sports, such as football and rugby, involve energy-sapping whole body tackles, scrummaging and wrestling for possession of the ball. Unfortunately, the energy cost of repeated body contact by players is technically difficult to obtain and so energy expenditures in these team sports is generally under-estimated because they are based only on players’ movements [3]. Furthermore, participation in tournaments requires players to compete more than once a day with only a few hours of recovery as is the case in, for example, field hockey and rugby sevens competitions [4].

There are several recent relevant reviews on carbohydrate and exercise [1, 5] as well as the recommended amounts of dietary carbohydrate that supports training and competition [6, 7]. How closely team sport athletes follow these recommendations has also been assessed [8]. The present brief review on carbohydrate intake on sport team performance is focussed largely on studies that use intermittent high-intensity running because of its relevance to the performances of team sport athletes.

## Energy Metabolism to Sustain High-Intensity Exercise

Our ability to exercise at high intensity depends on the capacity of our skeletal muscles to rapidly replace the adenosine triphosphate (ATP) used to support all energy-demanding processes during exercise. The two metabolic systems that generate ATP in skeletal muscle are described as ‘anaerobic’ and ‘aerobic’. To avoid misunderstanding about the function of these two energy systems, it is important to recognise that they work in concert not in isolation. For example, during a sprint the high rate of ATP production is provided by anaerobic energy metabolism while the physiological functions of the heart and other organs are supported by ATP derived from ongoing aerobic metabolism.

The anaerobic production of ATP is fuelled by the degradation of the intra-muscular stores of phosphocreatine (PCr) and glycogen, a glucose polymer. Skeletal muscle contains about five times more PCr than ATP and it is resynthesized by ongoing aerobic metabolism. Muscle glycogen, is degraded during contraction to generate ATP rapidly, but the process is accompanied by the production of lactate and hydrogen ions (for review see Girard et al. [9]). For example in a single 6-s sprint, glycogen degradation (glycogenolysis) contributes 50 % of the ATP production, whereas PCr contributes 48 % and the remaining 2 % is provided by the muscle’s small store of ATP [10].

The aerobic degradation of glycogen is a slower process than its anaerobic degradation; nevertheless it produces about 12 times more ATP (~36 mmol) than its anaerobic degradation. Even more ATP is produced by the oxidation of fatty acids (140 mmol). However, while aerobic metabolism generates more energy per unit of fuel than anaerobic metabolism, it is too slow to support the high rate of ATP turnover required during sprinting. Nevertheless, during recovery between sprints, aerobic metabolism is responsible for the re-synthesis of PCr as well as covering the energy cost of submaximal running. As the game progresses and the number of sprints increase, there is an even greater contribution of aerobic metabolism, especially during the lower intensity activities between sprints [11, 12]. The more economical use of glycogen as the activity continues is largely the result of an increase in aerobic production of ATP from glycogen, glucose and fatty acids.

Traditional endurance and high-intensity interval training increase the aerobic capacity of skeletal muscles that allows fatty acid oxidation to contribute to energy metabolism at higher exercise intensities than before training. It is now known that carbohydrate ingestion may be manipulated acutely around the training session to support the desired adaptation. For example, exercise following a low-carbohydrate diet has a marked influence on the expression of genes that promote an increase in fat metabolism [13]. Although an up-regulation of fatty acid oxidation will never cover the high demands for ATP re-synthesis required during sprints [14, 15], the oxidation of fat will play a supporting role during periods of recovery between repeated high-intensity efforts [16].

No single bodily system that is required to support the demands of team sport activity appears to be exclusively influenced by carbohydrate ingestion. For example, peripheral depletion of muscle glycogen in sub-cellular compartments such as the sarcoplasmic reticulum will influence the flux of calcium and impair the contractile property of the muscle [17, 18]. However, a diminished central drive associated with exercise-induced hypoglycaemia has been speculated to be directly related to a reduced delivery of glucose as a substrate to the brain [19]. Indeed, carbohydrate feedings are associated with enhanced perceived activation and a lowered perception of effort during intermittent running in comparison to the ingestion of placebo [20]. Thus, the main benefits of following a high-carbohydrate diet and ingesting carbohydrate during exercise are the availability of substrate for central and peripheral function.

## Exercise Protocols

For laboratory assessments to provide insight into the influence of dietary interventions on exercise performance, they should reproduce the demands of team sports that include acceleration, deceleration, as well as running at a range of speeds. This has typically been achieved by using intermittent, variable-speed shuttle running over a distance of 20 m [21, 22].

One such method is the Loughborough Intermittent Shuttle Running Test (LIST) that was designed to simulate the activity pattern characteristic of soccer and other stop-start sports [23]. The protocol consists of two parts: Part A is a fixed period of variable-intensity shuttle running over a distance of 20 m; Part B consists of continuous running, alternating every 20 m between 95 and 55 % of maximum oxygen uptake (*V*O_2_max) until volitional fatigue. Part A consists of five 15-min blocks of activity with a 3-min recovery between each block. In each 15-min block, runners complete the following cycle of activities: a walk, a sprint, a jog (55 % *V*O_2_max) and a run (95 % *V*O_2_max) and each cycle is repeated 11–12 times. An audible computer-generated ‘bleep’ helps participants maintain their predetermined pace with the exception of the sprints. Times for 15 m of the 20-m sprint are recorded using photo-electric timing gates. The physiological responses and distances covered during the 90-min LIST compare well with those recorded for professional soccer matches. This generic protocol provides an assessment of endurance running capacity during variable-speed running and also sprint performance. The protocol has been modified and adapted to include assessment of sport-specific fitness and in some cases sport-specific skills.

For example, Davis and colleagues modified the LIST protocol in an attempt to more closely resemble basketball by including two 15-min periods, with a 20-min ‘half-time’, then two additional 15-min periods, after which the players completed a shuttle run to fatigue [24]. This protocol also included measures of jumping ability and mental function. Subsequently, they extended this protocol to include a wider range of measures of both peripheral and central nervous system functions during the ‘half-time’ between each of the two 15-min quarters [25]. Afman and colleagues also adopted a modified version of the LIST to study the effects of nutritional interventions on basketball-specific skills as well as performance [26]. Rugby is a stop-and-go sport that includes set-piece contact of opposing players in the form of scrums as well as whole-body tackling. Although energy cost of scrummaging and tackles are difficult to measure, they are energy-sapping activities that have an accumulative influence on players’ performance. Roberts and colleagues have validated a performance test that is based on the LIST protocol and includes simulated scrummaging and tackling [27].

It is important to acknowledge that in these studies the exercise intensity is prescribed with only the sprint speeds being self-selected, whereas in competitive games the players pace themselves. This limitation has recently been addressed by Ali and colleagues with the addition of “self-paced” sections to the LIST protocol [28]. In this modification, games players complete four 15-min blocks of the standard LIST protocol during which the intensity of the cycle of activities of the first two blocks were dictated by an audible computer-generated bleep whereas during the last two 15 blocks the exercise intensities of the activity cycle were self-selected. This modification was introduced to improve the ecological validity of the protocol [29]. The LIST protocol and its modifications is essentially a method of assessing both endurance capacity (time to fatigue) and performance (sprint times) of games players after a prolonged period of intermittent variable-speed running. However, it is not skill specific to any one stop-start sport.

Recent studies have adopted and modified the LIST protocol to evaluate the performance benefits of nutritional interventions on sports-specific skills, as well as performance [30–35]. In the development of the Copenhagen Soccer Test, Bangsbo and colleagues included a full range of soccer-related activities in addition to the assessment of running performance [34]. More relevant to the current review is that they showed that completion of 60 min of the Copenhagen Soccer Test reduces muscle glycogen levels to similar values as those recorded during competitive soccer matches. Following a competitive soccer match muscle glycogen stores are reduced by 50–60 % of pre-match values. It should be noted that the loss of glycogen during intermittent variable running is not even across both type 1 and type 2 fibres [34, 36].

Early studies of work rates during soccer matches revealed the link between muscle glycogen stores and activity patterns of players: those players with low pre-match glycogen levels covered less ground than those with high values [37, 38]. Therefore, it is not surprising that team sport players are encouraged to restock their carbohydrate stores before competition as well as during recovery between training sessions [6]. A well-established method of restocking carbohydrate stores involves reducing training loads whilst in parallel increasing the amount of carbohydrate in the diet [39]. Although there are several seminal running and cycling studies that show the benefits of undertaking exercise with well-stocked glycogen stores, there are fewer studies on the performance advantages in stop-start team sports.

## Pre-Exercise Nutrition

Balsom and colleagues showed the positive impact of carbohydrate loading on the performance of multiple cycling sprints [11]. They extended their study to examine the influence of carbohydrate loading on the performances of six soccer players during a 90-min four-a-side soccer match [40]. Muscle glycogen levels were lowered 48 h earlier when players completed a variable-speed shuttle-running test. Thereafter, they changed the carbohydrate content of their diet to one with either 65 or 30 % of daily energy intake. After the high-carbohydrate diet, muscle glycogen level was 28 % higher than after the low-carbohydrate diet. Analysis of the movement patterns during the simulated four-a-side soccer match showed that after the high-carbohydrate diet, players performed 30 % more high-intensity running than after the low-carbohydrate pre-match diet. There was no difference between the performances of technical skills during the four-a-side matches following the two dietary preparations [40]. It is important to note that movement patterns during competitive team games have a high day-to-day variability [41].

The well-entrenched recommendation to eat an easy-to-digest high-carbohydrate meal about 3 h before exercise does not usually include mention of the type of carbohydrate [1]. Nevertheless, it is assumed that they are high-glycaemic index (HGI) carbohydrates that are digested and absorbed more quickly than low-glycaemic (LGI) index carbohydrates. Eating a HGI carbohydrate meal, that provided 2.5 g/kg body mass (BM), 3 h before exercise, increases muscle glycogen levels by about 11–15 % [42, 43]. This relatively modest increase in muscle glycogen is a consequence of the early removal of systemic glucose by the liver and 3 h is insufficient for the digestion and absorption of the carbohydrate meal. In contrast, when an energy-matched LGI carbohydrate meal was consumed there was no measureable increase in muscle glycogen levels. It is reasonable to assume that the slower digestion and absorption of the high-fibre carbohydrate meal results in a delayed delivery of glucose to the systemic circulation and hence skeletal muscles [42]. During subsequent submaximal treadmill running, there was a lower rate of carbohydrate oxidation and a higher rate of fat oxidation than when runners consumed the HGI pre-exercise meal. The lower rate of carbohydrate oxidation suggests that muscle glycogen stores were used more sparingly, i.e. glycogen sparing. When the endurance-running capacity of treadmill runners were compared following consuming pre-exercise HGI and LGI carbohydrate meals on separate occasions, the time to fatigue was greater following the LGI meal [44].

Consuming a LGI carbohydrate pre-exercise meal results in a smaller rise in plasma insulin level than is the case following HGI carbohydrate meals. As a consequence, the inhibition of fatty acid mobilisation is reduced, the rate of fat metabolism during subsequent exercise is increased, and so muscle glycogen is oxidised more slowly. This more economic use of the limited glycogen stores is an advantage during prolonged submaximal exercise; however, brief periods of sprinting rely on a high rate of glycogenolysis and phosphocreatine degradation. Therefore, as mentioned previously even a higher rate of fat metabolism, following a LGI carbohydrate meal, cannot provide ATP fast enough to support high-intensity exercise. Therefore, it is not surprising that the few studies that compared the impact of HGI and LGI carbohydrate pre-exercise meals on performance during intermittent brief high-intensity exercise failed to show differences [45–47].

When considering the merits of HGI and LGI pre-exercise meals it is important to remember that to achieve the same amount of carbohydrate and energy, the LGI meal will have a greater amount of food than in the HGI meal [47]. The reason for this is that LGI carbohydrates generally have higher fibre content and so more food has to be consumed to match the amount in HGI foods. The higher fibre content of LGI carbohydrate foods results in earlier satiation than following the consumption of HGI carbohydrate foods. One consequence is that athletes may consume less carbohydrate when recommended to eat LGI foods and so do not sufficiently restock their glycogen stores.

## Nutrition During Exercise

During high-intensity exercise, the permeability of the muscle membrane to glucose is sensitised via a multitude of signalling pathways thought to include adenosine monophosphate kinase and calcium amongst many others [48]. However, the delivery of glucose to the muscle is reliant on adequate perfusion of skeletal muscle capillaries while maintaining overall plasma glucose levels [49]. The benefits of ingesting a carbohydrate-electrolyte (CHO-E) solution during endurance exercise are well established [50]. Less attention has been paid to prolonged intermittent exercise, though early speculation suggested improvements in performance would be similar [51]. In pursuit of answers to these questions, Nicholas and colleagues undertook a study in which they provided games players with either a 6.5 % CHO-E solution or a taste- and colour-matched placebo solution between the 15-min activity blocks of the LIST. After performing 75 min of the LIST, the games players completed Part B, i.e. alternated 20-m sprints with jogging recoveries to fatigue. Ingesting the CHO-E solution resulted in a 33 % greater running time, i.e. beyond the five blocks of the LIST, than when they ingested the placebo [52]. A similar result was obtained by Davis and colleagues using a modified form of the LIST to examine the influences of ingesting a 6 % CHO-E solution with and without chromium supplementation on endurance intermittent shuttle-running performance. Ingesting a 6 % CHO-E solution improved shuttle-running time by 32 % compared with ingesting a placebo, but there was no added benefit from including chromium with the CHO-E [53].

Davis and colleagues modified the LIST protocol to more closely resemble the activity periods in basketball. They included four 15-min blocks (quarters) of variable-speed running, walking and vertical jumps with a 20-min resting ‘half-time’ between the second and third quarters [24]. At the end of the fourth quarter, the players completed 20-m intervals of shuttle running alternating between 120 and 55 % *V*O_2_max to voluntary fatigue. In the brief rest periods between each 15-min block, the games players also completed a set of mental and physical tests, namely: vertical jumps, a modified hop-scotch test to assess whole body motor skill, and mental function tests, i.e. Stroop colour word test as well as completing a Profile of Mood States questionnaire. The games players ingested immediately before and throughout exercise either a 6 % CHO-E solution or a taste- and colour-matched placebo. Shuttle running time to fatigue was 37 % greater when the players ingested the CHO-E solution and sprint times were better maintained during the last quarter than during the placebo trial. This study was later repeated with a larger number of male and female games players using the same methods to examine the impact of ingesting a 6 % CHO-E solution in relation to a taste-matched placebo. They included measures of peripheral and CNS function during the basketball-related exercise protocol and found faster 20-m sprint times, enhanced motor skills and improved mood state during the last quarter when the games players ingested the CHO-E solution [25].

Afman and colleagues also adopted a similar modification of the LIST protocol and included a ‘lay-up’ shot as a measure of basketball-specific skill [26]. In contrast to the results reported by Davis and colleagues, they found no performance benefit when their basketball players ingested 75 g of sucrose in 500 mL of orange juice 45 min before they completed the basketball test. In the sucrose trial, players were hypoglycaemic at the end of the first quarter and their performance of the ‘lay-up’ shot and sprinting speeds were significantly poorer than at the same time during the placebo trial. However, during the fourth-quarter, sprint performance was not different from those on the placebo trial [26]. The ingestion of the large bolus of sucrose 45 min before exercise is known to cause hypoglycaemia at the onset of exercise but without a detriment to endurance-running capacity [54].

In a three-trial study, Stokes and colleagues examined the performance benefits of ingesting a CHO-E solution and a CHO-E solution with caffeine in comparison with a placebo solution during a rugby performance test [35]. They reported that there were no significant differences in the results of the performance tests, which were embedded in their shuttle-running protocol. Nevertheless, they predicted with 98 % confidence that ingestion of the carbohydrate-caffeine mixture would result in a 2 % improvement in overall performance and concluded that the combination of carbohydrate and caffeine was superior to carbohydrate alone and the placebo solution.

In an attempt to identify the level of CHO-E solutions that is most effective in improving performance of adolescents during variable-speed shuttle running, Phillips and colleagues examined the influences of 2, 6 and 10 % CHO-E solutions [55]. Seven young team games players (five boys and two girls: average age of 13.3 years) performed the LIST protocol after an overnight fast on three occasions during which they ingested the same volume of the CHO-E solutions. They found that endurance capacity on the 6 % CHO-E trial was significantly greater (34 %) than on the 10 % CHO-E trial; however, there were no sprint performance differences between the 2, 6 and 10 % CHO-E trials. Furthermore, there was a trend for the performances on the 2 % CHO-E solution trial to be better than on the 10 % CHO-E solution trial. However, it would be unwise to extrapolate the results of this study to adolescents per se because the participants were an uneven number of boys and girls [55].

Foskett and colleagues addressed the question of whether or not ingesting a CHO-E solution during prolonged, intermittent high-intensity shuttle running has performance benefits for games players when their muscle glycogen stores were well stocked before exercise [56]. To test this hypothesis, six university-level soccer players completed six blocks of the LIST (90 min) and then consumed a high-carbohydrate diet for 48 h before repeating the LIST to fatigue. Carbohydrate loading increased muscle glycogen levels by about 50 % more than the normal values for these games players. During subsequent performance of the LIST, they ingested either a 6.5 % CHO-E solution or a coloured-taste matched placebo throughout exercise. At the end of 90 min, the games players, running with a ‘fitness- matched’ partner, continued to complete the standard 15-min block of activities to the point of fatigue. The total exercise time during the CHO-E trial was significantly longer (158 min) than during the placebo trial (131 min) [56]. There was no evidence of glycogen sparing and yet during the CHO-E trial the soccer players ran for an additional 27 min beyond their performance time during the placebo trial. While only speculative, the greater endurance may have been a consequence of higher blood glucose levels that did not compromise the supply of glucose to the central nervous system as early as in the placebo trial, thus delaying an inhibition of motor drive as glycogen stores became ever lower [57, 58].

There is some evidence that gastric emptying of a CHO-E solution is slower while performing brief periods of high-intensity cycling than during lower intensity exercise [59]. To examine whether or not the same slowing of gastric emptying occurs during variable-speed running, Leiper and colleagues completed two studies in which games players ingested CHO-E solutions before and during exercise [60, 61]. In the first study, they monitored gastric emptying of a 6 % CHO-E solution before and after two 15-min periods of play during an indoor, competitive five-a-side soccer match. The same gastric emptying and timing was repeated while the soccer players performed two 15-min periods of walking with the same 10-min rest between the two activity periods. Gastric emptying was slower during the first 15-min period than during the walking-only trial, but during the second 15 min of the soccer game there was no statistical difference in the emptying rate. In total, the volume of fluid emptied from the stomach was less than during the same period while walking [60]. In the second running study, gastric emptying of a 6.4 % CHO-E solution and a CHO-free solution were monitored before and after two 15-min periods of the LIST and also after two 15-min periods of walking. The exercise intensities during the two 15-min activity cycles of the LIST were higher and more closely controlled than those self-selected exercise intensities achieved during the five-a-side soccer game. Nevertheless, the results were quite similar in that gastric emptying was slower during the first 15 min of exercise both for the CHO-E and the placebo solutions than while walking for the same period. However, during the second 15 min, gastric emptying of both solutions was similar during both the running and the walking trials with a trend for slightly faster emptying rates [61]. Whether or not this greater gastric emptying later in exercise suggests an acute adaptation to coping with large gastric volumes remains to be determined. Even with an intensity-induced reduction in gastric emptying, the available evidence does not suggest that team sport players should drink carbohydrate-free solutions. On the contrary, there is sufficient evidence to support the ingestion of CHO-E solutions during prolonged, intermittent variable-speed running to improve endurance capacity [24, 52, 55]. However, even recognising the benefits of ingesting CHO-E solutions during intermittent variable-speed running, young athletes appear to not meet the recommended intakes [8].

Carbohydrate gels provide a convenient means of accessing this essential fuel during prolonged running and cycling. However, there are only a few studies on the benefits of ingesting carbohydrate gels during variable-speed shuttle running. Of the two available studies, both report that ingesting carbohydrate gels improves endurance running capacity. One of the studies reported that when games players ingested either an isotonic carbohydrate gel or an artificially sweetened orange placebo while performing the LIST protocol, their endurance capacity was greater during the gel (6.1 min) than during the placebo (4.2 min) trial [62]. In the second study on intermittent shuttle running, Phillips and colleagues compared the performances of games players when they ingested either a carbohydrate gel or non-carbohydrate gel before and at 15-min intervals while completing the LIST protocol [63]. They reported that during the carbohydrate-gel trial, the games players ran longer in Part B (4.6 min) than during placebo trial (3.8 min) but there were no differences in sprint speeds. Concerns about the potential delay in gastric emptying when ingesting carbohydrate gels before and during exercise are allayed by the performance benefits reported in the above studies. In addition, it appears that the rate of oxidation of carbohydrate gels during 180 min of submaximal cycling is no different to that after ingesting a 12.5 % CHO-E solution [64].

Although carbohydrate-protein mixtures have mainly been considered as a means of accelerating post-exercise glycogen re-synthesis, Highton and colleagues examined their performance benefits during prolonged variable-speed shuttle running [65]. Using a modified version of the LIST in which the games players self-selected their own speeds during the final two 15-min blocks of activity, they reported that there was trend for a better performance following the ingestion of the carbohydrate-protein mixture (6 % CHO + 2 % whey protein) than following the ingestion of a carbohydrate solution (8 %). However there were no significant differences in the performance between trials.

## Exercise in Hot Environments

Exercise performance in the heat is generally poorer than during exercise in temperate climates. Team sports are no exception, for example Mohr and colleagues have clearly shown that the performance of elite soccer players is significantly compromised when matches are played in the heat, i.e. 33 °C [66]. There are only a few studies on exercise performance during variable-speed running in hot and cooler environments. Morris and colleagues assessed the exercise performances of university student games players performing five 15-min blocks of the LIST (Part A) followed by intermittent running and resting (60-s run at 99 % *V*O_2_max and 60-s rest) to fatigue in an environment of 30 and 20 °C [67]. Only 7 of the 12 games players were able to complete both parts of the LIST in the hot environment and the total distance covered was 21 % less and 15-m sprint times were slower than in the cool environment. There was a strong correlation between the rate of rise of rectal temperature and distance covered (*r* = − 0.94) in the heat but not during exercise in the cooler environment.

Using the same experimental design, Morris et al. [67] examined the performance of 16 well-trained female athletes (eight games players and eight endurance runners) undertaking variable-speed intermittent exercise in hot (30 °C) and cool (16 °C) environments. There were no differences in the distance covered by the games players and endurance runners but the total distance was 25 % less in the hot compared with the cooler environment. This 25 % reduction in endurance performance in the heat is similar to the 21 % reported for male games players performing the same intermittent exercise protocol [67]. The 15-m sprint speeds of the female athletes were also significantly slower in the heat, declining with test duration, which was not the case during exercise in the cooler environment. Again, there was a high correlation between the rates of rise of the rectal temperatures of the athletes in the heat but it was less strong during exercise at the lower ambient temperature.

In a follow-up study, Morris et al. [68] examined whether or not the amount of glycogen used was greater during exercise in the heat (33 °C) compared with exercise in a cooler (17 °C) environment. Distance covered during the LIST protocol was 49 % less during exercise in the heat and average 15-m sprint times were slower than during exercise in the cooler environment. Rectal and muscle temperatures were significantly higher at the point of fatigue after exercising in the heat. Analyses of muscle biopsy samples taken from eight sportsmen before and after completing the LIST protocol under the two environmental conditions showed that the rate of glycogenolysis was greater in seven of the eight men in the heat. However, glycogen levels were higher at fatigue after exercise in the heat than after exercise in the cooler environment [68]. Muscle glycogen and blood glucose levels were lower at exhaustion during exercise in the cooler environment, suggesting that reduced carbohydrate availability contributed to the onset of fatigue. At exhaustion after exercise in the heat muscle, glycogen and blood glucose levels were significantly higher, suggesting that fatigue was largely a consequence of high body temperature rather than carbohydrate availability.

Endurance capacity during exercise in the heat is improved when sufficient fluid is ingested [69], but does drinking CHO-E solution rather than water have added performance benefits? This question was addressed in a three-trial design in which nine male games players ingested either a flavoured-water placebo, a taste-matched placebo, or a 6.5 % CHO-E solution in random order while performing in a hot environment (30 °C) [70]. Although ingesting the CHO-E solution resulted in greater metabolic changes, there were no differences in the performances during the three trials. While the games players were accustomed to performing prolonged variable-speed running during training and competition, they were not acclimatised to exercising in the heat. This lack of acclimatisation was clearly reflected by a 19 % improvement in performance between the first and third trials, irrespective of treatment.

Clarke and colleagues attempted to tease out the benefits of delaying the rise in core temperature and CHO-E ingestion on performance in the heat [71]. Twelve male soccer players completed two 45-min periods, with a 15-min break between each ‘half’, of an intermittent, high-speed treadmill running test, based on the activity patterns common in soccer, in an environment of 30.5 °C and 44 % relative humidity. The four-trial design included two trials in which the soccer players were pre-cooled before the test and two trials without pre-cooling. In each pair of trials, the soccer players ingested, at 15-min intervals, either a 6.6 % CHO-E solution or a colour- and flavour-matched placebo. Performance was assessed at the end of 90 min at the self-selected speed that the soccer players predicted was sustainable for 30 min but ran for only 3 min at this speed. Thereafter, their high-intensity exercise capacity was determined during uphill treadmill running that was designed to lead to exhaustion in about 60 s [72]. They found that pre-cooling and CHO-E solution ingestion resulted in a superior performance at the self-selected running speed than CHO-E ingestion alone. However, CHO-E solution ingestion, with or without pre-cooling, resulted in a longer running time, albeit quite short, during high-intensity exercise test than during the placebo trials. The findings of this study provide evidence to support the conclusion that variable-speed running in hot environments is limited by the degree of hyperthermia before muscle glycogen availability becomes a significant contributor to the onset of fatigue.

## Recovery

Consuming carbohydrates immediately after exercise increases the repletion rate of muscle glycogen [73]. In competitive team sports, the relevant question is whether or not this nutritional strategy also returns performance during subsequent exercise. Addressing this question, Nicholas and colleagues recruited games players who performed five blocks of the LIST (75 min) followed by alternate 20-m sprints with jogging recovery to fatigue, and 22 h later they attempted to repeat their performance [74]. During the recovery they consumed their normal diet with either additional carbohydrate to achieve a total intake of 9 g/kg BM or their normal carbohydrate intake (5 g/kg BM) plus extra protein so as to match energy intake of the carbohydrate diet. After the high-carbohydrate recovery diet, the games players were able to match their previous day’s performance. In contrast when they consumed their normal amount of carbohydrate, and an equal energy intake, the players failed to reproduce their previous day’s performance.

When this study was repeated using energy- and macro-nutrient-matched HGI and LGI carbohydrate meals during the 24-h recovery, there were no differences in performance of the games players [47]. This is not surprising because the advantage of pre-exercise LGI carbohydrate meals is the lower plasma insulin levels that allow greater rates of fat mobilisation and oxidation, which in turn benefit low- rather than high-intensity exercise.

Clearly providing carbohydrates during recovery from exercise accelerates glycogen re-synthesis as does the degree of exercise-induced depletion [75]. It also appears that the environmental conditions may influence the rate of glycogen re-synthesis. When nine male individuals cycled for an hour to lower muscle glycogen and then consumed carbohydrate (1.8 g/kg BM) during a 4-h recovery at room temperature (22.2 °C) and in a hot environment (32.6 °C), the rate of re-synthesis was slower at the higher ambient temperature [76]. Recovery in a cool environment (7 °C) does not slow the rate of muscle glycogen re-synthesis [77]. In contrast, local cooling of skeletal muscle, a common recovery strategy in team sport, has been reported to have either no impact on or delay glycogen re-synthesis [78]. Clearly, further research is required.

It has been suggested that adding protein to carbohydrate during recovery increases the rate of glycogen re-synthesis and so improves subsequent exercise capacity. The rationale behind this suggestion was that a protein-induced increase in plasma insulin level will increase the insulinogenic response to consuming carbohydrate leading to a greater re-synthesis of muscle glycogen [79]. Although a greater rate of post-exercise glycogen re-synthesis and storage has been reported following the ingestion of a carbohydrate-protein mixture compared with a carbohydrate-matched solution, there were no differences in plasma insulin responses [80]. Nevertheless, more recent studies suggest that ingesting sufficient carbohydrate (~1.2 g/kg BM) during post-exercise recovery can maximise the rate of glycogen storage and improve subsequent, treadmill running endurance capacity more than is seen following the ingestion of an energy-matched carbohydrate-protein mixture [81]. The possibility of enhancing glycogen storage after competitive soccer matches by consuming meals high in whey protein and carbohydrate has recently been explored by Gunnarsson and colleagues [82]. They fed two groups of elite soccer players meals of similar energy content (En) but were either high in carbohydrate (70 % En) and whey protein (21 % En) and low in fat (8 % En) or meals containing their normal amount of carbohydrate (55 % En), protein (18 % En) and fat (26 % En) for 48 h following a competitive soccer match. In the carbohydrate-protein group (*n* = 9), the whey protein was consumed as a supplement immediately after the match and then with the three meals per 24 h. After the 48-h dietary intervention, there were no differences in muscle glycogen storage between the carbohydrate-whey protein and control groups [82]. While post-exercise carbohydrate-protein mixtures may not enhance glycogen storage or enhance subsequent exercise capacity, they promote skeletal muscle protein synthesis [83].

## Conclusions

Prolonged periods of multiple sprints drain muscle glycogen stores, leading to a decrease in power output and a reduction in the general work rate during training and competition. Adopting nutritional strategies to ensure that muscle glycogen stores are well stocked prior to training and competition helps delay fatigue. There is now clear evidence for the following recommendations. (1) On the day of training or competition, pre-exercise meals should contain HGI carbohydrate foods rather than LGI carbohydrate foods because of the more rapid delivery of glucose to liver and muscle via the systemic circulation. (2) During participation in multiple-sprint sports, ingesting a well-formulated CHO-E solution or gel improves endurance capacity and may prevent a significant decay in sprint speeds. (3) Consuming HGI carbohydrates immediately after exercise accelerates the rate of glycogen re-synthesis, which is essential for players training or competing on successive days.
